# Impaired fasting blood glucose is associated to cognitive impairment and cerebral atrophy in middle-aged non-human primates

**DOI:** 10.18632/aging.101148

**Published:** 2016-12-28

**Authors:** Fathia Djelti, Marc Dhenain, Jérémy Terrien, Jean-Luc Picq, Isabelle Hardy, Delphine Champeval, Martine Perret, Esther Schenker, Jacques Epelbaum, Fabienne Aujard

**Affiliations:** ^1^ MECADEV UMR 7179, Centre National de la Recherche Scientifique, Muséum National d'Histoire Naturelle, 91800 Brunoy, France; ^2^ Université Sorbonne Paris Cité, 75013 Paris, France; ^3^ Centre National de la Recherche Scientifique, Université Paris Sud, Université Paris Saclay, UMR 9199, Neurodegenerative Diseases Laboratory, 92265 Fontenay-aux-Roses, France; ^4^ Commissariat à l'Energie Atomique et aux Energies Alternatives (CEA), Direction de la Recherche Fondamentale (DRF), Institut d'Imagerie Biomédicale (I2BM), MIRCen, 92265 Fontenay-aux-Roses, France; ^5^ Laboratoire de Psychopathologie et de Neuropsychologie, EA 2027, Université Paris 8, 93200 Saint-Denis, France; ^6^ Institut de Recherches Servier, 78290 Croissy-sur-Seine, France; ^7^ Centre Psychiatrie & Neurosciences, UMR S894 INSERM, Université Paris Descartes, 75014 Paris, France

**Keywords:** blood glucose, middle age, spatial memory performance, hippocampal/septal atrophy, spontaneous models, *Microcebus murinus*

## Abstract

Age-associated cognitive impairment is a major health and social issue because of increasing aged population. Cognitive decline is not homogeneous in humans and the determinants leading to differences between subjects are not fully understood. In middle-aged healthy humans, fasting blood glucose levels in the upper normal range are associated with memory impairment and cerebral atrophy. Due to a close evolutional similarity to Man, non-human primates may be useful to investigate the relationships between glucose homeostasis, cognitive deficits and structural brain alterations. In the grey mouse lemur, *Microcebus murinus*, spatial memory deficits have been associated with age and cerebral atrophy but the origin of these alterations have not been clearly identified. Herein, we showed that, on 28 female grey mouse lemurs (age range 2.4-6.1 years-old), age correlated with impaired fasting blood glucose (r_s_=0.37) but not with impaired glucose tolerance or insulin resistance. In middle-aged animals (4.1-6.1 years-old), fasting blood glucose was inversely and closely linked with spatial memory performance (r_s_=0.56) and hippocampus (r_s_=−0.62) or septum (r_s_=−0.55) volumes. These findings corroborate observations in humans and further support the grey mouse lemur as a natural model to unravel mechanisms which link impaired glucose homeostasis, brain atrophy and cognitive processes.

## INTRODUCTION

With the progressive aging of the World population, an increasing number of people suffer from cognitive impairment and dementia. These deficits affect quality of life and cause social, economic and public health problems [1–3]. A clear relationship has been established between age-associated cognitive decline and selective regional brain atrophy from normal aging to dementia [4–5].

Cognitive decline is not homogeneous in humans and the determinants leading to differences between subjects are not fully understood. Middle-aged metabolic disorders [6–8] culminating in type 2 diabetes (T2D) [9–11] could be a risk factor to memory impairment and cerebral atrophy. T2D is characterized by hyper-glycaemia (> 7mmol/L), impairment of glucose tolerance leading to insulin resistance [12]. Patients with type 2 diabetes display memory impairment [9–11] and anatomic brain alterations including hippocampus and amygdala atrophy [13]. A phylogenetic study of the longevity of 16 rodent species even concluded that modulation of IGF1R signaling in nervous tissue, but not in the peripheral tissues, is an important factor in the evolution of longevity in mammals [14].

Intermediate states between normal glucose homeostasis and diabetic hyperglycaemia are currently described in aging. They are characterized either by impaired fasting glycaemia (IFG) or by impaired glucose tolerance (IGT) [15]. IFG occurs when fasting blood glucose level is consistently elevated above what is considered normal levels. IGT evaluates how quickly glucose is cleared from the blood by testing glycaemia during two-hours after ingestion of a standard dose of glucose. These two states are associated with an increased risk for various pathologies including cardiovascular pathologies. Non diabetic subjects with IFG display mild cognitive impairment and regional brain atrophy [16–20]. In laboratory rodents, in which diabetic stages are induced by chemicals or diet, experimental studies also demonstrate a relationship between glucose dys-regulation and memory performance [21–22]. However, these models are far from recapitulating the same pathophysiology as observed in Man [23–24].

In contrast to rodent models, the grey mouse lemur (*Microcebus murinus*), a non human primate, could represent an interesting alternative to study the relationships between preclinical glucose dys-homeostasis, cerebral atrophy and cognitive impairment. Firstly, this small animal (12cm, 60-100g) has a relatively short maximum lifespan (approximately 12 years in captivity) in comparison to other primates, which have a much longer lifespan [25]. Secondly, as a primate, it shares several genetic, physiological and anatomical similarities with Man [25–26]. Thirdly, a large inter-individual heterogeneity of spatial memory performance is observed in aging grey mouse lemurs [27]. Fourthly, atrophy of distinct brain regions (cortical regions, hippocampus, caudate nucleus), including atrophy of some hippocampal subfields is observed in aged mouse lemurs [28]. Finally, in old animals, cognitive defects correlate with hippocampus and septum atrophy [29] but the origin of these alterations is still partly unknown.

In the present study, blood glucose measures were determined in young and middle-aged mouse lemurs. A correlation between fasting blood glucose and age was evidenced. A majority (56%) of middle-aged animals displayed high normal value of fasting blood glucose levels whereas the remaining ones (44%) presented a range of values equivalent to young animals. Impaired fasting blood glucose was associated with memory alterations and hippocampus/septum atrophy. These results emphasize the critical impact of early glucose dys-homeostasis on morphological brain alterations and on cognitive function.

## RESULTS

### Glucose metabolic measures in mouse lemurs

**Figure 1:**
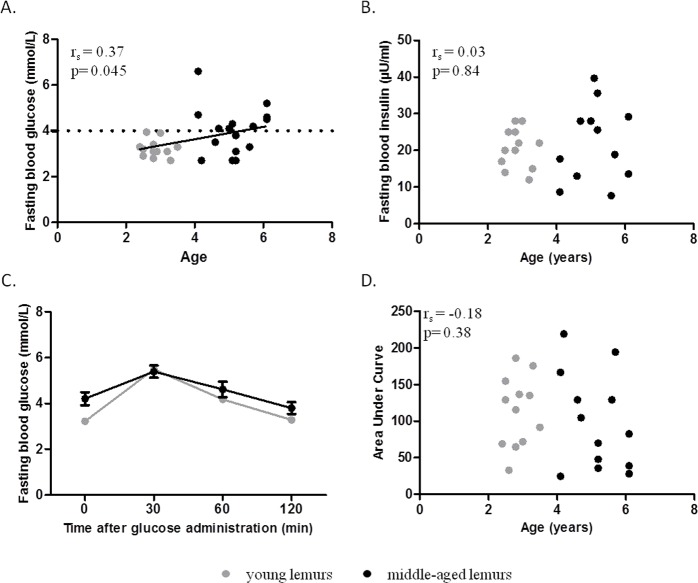
Relationship between glucose homeostasis parameters and age Glucose metabolism parameters were evaluated in young (grey symbols, range: 2.4 to 3.5 years old) and middle-aged (black symbols, range: 4.1 to 6.1 years old) mouse lemurs. (**A**) Spearman correlation of fasting blood glucose levels and age. (**B**) Spearman correlation of fasting blood insulin levels and age. (**C**) Oral glucose tolerance test (OGTT): Blood glucose was measured 0, 30, 60 and 120 min after oral administration of 1.75g glucose/g of body mass (Two-way ANOVA: age p<0.01, time p<0.001, time × age p=0.11). (**D**) Spearman correlation of area under curve of OGTT and age.

Indicators of glucose metabolism were evaluated in female mouse lemurs aged from 2.4 to 6.1 years old (n=28). A correlation occurred between age and fasting blood glucose (r_s_= 0.37, p=0.045, Figure 1A) but fasting blood insulin levels and HOMA-IR index (an index of insulin resistance) were not significantly correlated with age (fasting insulin, r_s_=0.03, p=0.84; HOMA-IR: r_s_=0.22, p=0.28; Figure 1B, Table 1). Oral glucose tolerance was similar between animals, independently of age (Figure 1C) and so the glucose tolerance index was not linked to age (r_s_=−0.18, p=0.38; Figure 1D, Table 1).

**Table 1:** Spearman correlation rank coefficients between physical, glucose homeostasis parameters and number of errors in the Barnes maze

		Age	Body mass	Fasting blood glucose	Fasting insulin	HOMA-IR	Glucose tolerance index
Body mass	All	0.22					
	Young	0.11					
	Middle-aged	−0.15					
Fasting glucose	All	0.37*	0.08				
	Young	−0.15	0.37				
	Middle-aged	0.09	−0.12				
Fasting insulin	All	0.03	−0.09	0.02			
	Young	0.07	0.47	0.37			
	Middle-aged	0.09	−0.44	−0.11			
HOMA-IR	All	0.22	0.03				
	Young	0.08	0.50				
	Middle-aged	−0.06	−0.44				
Glucose tolerance index	All	−0.18	−0.20		−0.18	−0.21	
	Young	0.22	−0.25		−0.29	−0.44	
	Middle-aged	−0.24	−0.11		−0.05	0.09	
Number of errors	All	0.49**	−0.10	0.38*	−0.11	−0.09	−0.13
	Young	0.28	−0.24	−0.36	0.25	0.05	0.31
	Middle-aged	0.36	−0.17	0.56*	−0.23	−0.06	−0.31

* p<0.05

** p<0.001

HOMA-IR: Homeostasis model assessment of insulin resistance.

Animals were then dissociated into young (range: 2.4 to 3.5 years old, n=12) and middle-aged (range 4.1 to 6.1 years old, n=16) lemurs (Table 2). Body mass was not significantly different between the two groups (Table 2). Mean fasting blood glucose in young animals was 3.2±0.1 mmol/L (Table 2). Middle-aged animals presented significantly greater fasting blood glucose levels (4.0±0.3 mmol/L, p=0.03) associated with a higher inter-individual variability (range: 2.7-6.6 mmol/L, Table 2). Interestingly, two groups of middle-aged animals could be differentiated based on their fasting blood glucose. Indeed, 56% of middle-aged animals displayed a higher fasting glycaemia than any young animal (i.e. > 4 mmol/L) (Figure 1A).

**Table 2:** Characteristics of mouse lemurs

	Young	Middle-aged	p
Number	12	16	
Age (years)	**2.9 ± 0.1 (2.4 to 3.5)**	**5.1 ± 0.2 (4.1 to 6.1)**	**<0.0001**
Body mass (g)	82 ± 4 (65 to 108)	90 ± 4 (68 to 110)	0.16
Glucose homeostasis Fasting blood glucose (mmol/L)	**3.2 ± 0.1 (2.7 to 3.9)**	**4.0**± **0.3 (2.7 to 6.6)**	**0.03**
Fasting Insulin (μU/ml)	21 ± 2 (12 to 28)	22 ± 3 (8 to 40)	0.98
HOMA-IR index	3.0 ± 0.3 (1.6 to 4.8)	3.9 ± 0.5 (1.1 to 7.6)	0.17
Glucose tolerance index (AUC in mmol/L.min)	114 ± 14 (33 to 186)	98 ± 18 (25 to 219)	0.4
Spatial memory performance Number of errors	3.1 ± 0.3 (1 to 5)	5.1 ± 0.7 (2 to 11)	0.08

Data are Mean ± SEM (Range): HOMA-IR: Homeostasis model assessment of insulin resistance. AUC: Area Under Curve of Oral Glucose Tolerance Test.

### Spatial memory performance in mouse lemurs

**Figure 2:**
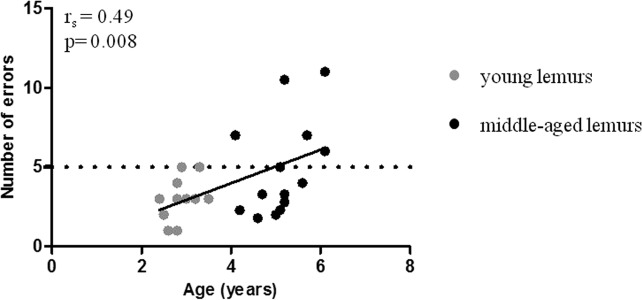
Relationship between spatial memory performance and age Spatial memory performance as reflected by the number of errors was determined with Barnes maze test and was measured in young (grey symbols, range: 2.4 to 3.5 years old) and middle-aged (blacik symbols, range: 4.1 to 6.1 years old) mouse lemurs. Spearman correlation of error number and age. The horizontal dotted line illustrates the threshold differentiating good and poor performers.

A Barnes maze task was used to evaluate spatial memory performances in mouse lemurs [27]. Number of errors correlated with age (r_s_=0.49, p=0.008; Figure 2, Table 1). Young animals displayed on average 3.1±0.3 errors during the test (Table 2) whereas the mean number of errors in middle-aged animals was 5.1±0.7 (Table 2). High inter-individual heterogeneity in middle-aged animals (range: 2-11 errors) suggested a discrimination in two groups. Forty four per cent of middle-aged animals presented a similar number of errors as compared with young animals whereas 56% displayed an increased number of errors (Figure 2). A threshold of 5 errors discriminated the two subpopulations of good and poor performers.

### Relationships between impaired fasting blood glucose and memory performance

**Figure 3:**
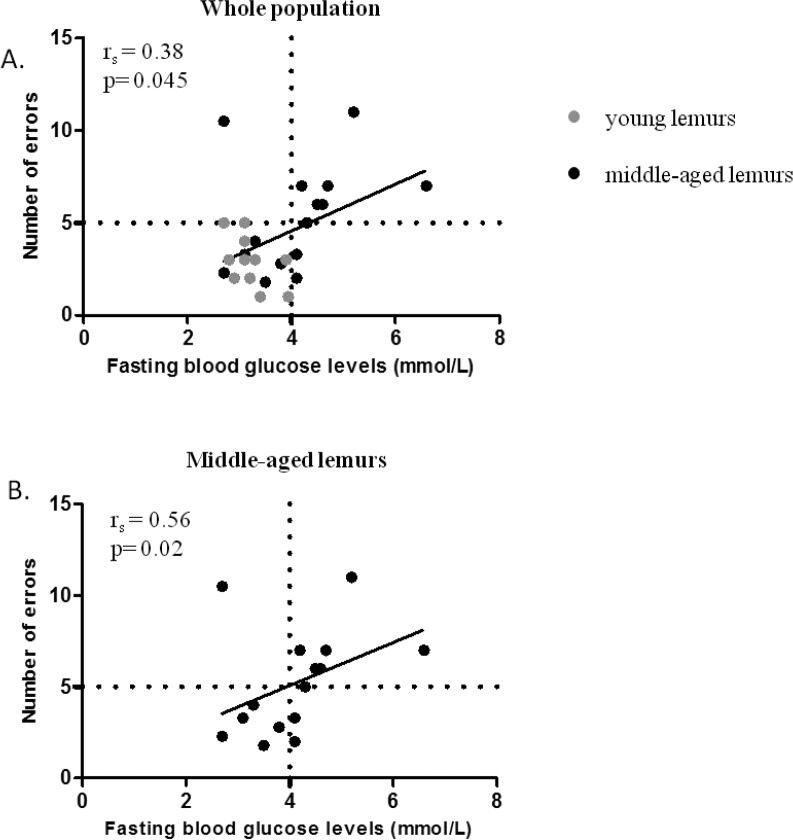
Relationship between fasting blood glucose and spatial memory performance (**A**) Spearman correlation between fasting blood glucose levels and number of errors in all lemurs (young in grey symbols and middle-aged in black symbols). (**B**) Spearman correlation between fasting blood glucose levels and number of errors in middle-aged lemurs only. Horizontal and vertical dotted lines illustrate the threshold differentiating good and poor performers in spatial memory test or high and normal blood glucose levels, respectively.

The number of errors and fasting glucose levels were significantly correlated in the whole population (r_s_=0.38, p=0.045, Figure 3A) and further so in middle-aged animals (r_s_=0.56, p=0.02, Figure 3B) but the correlation did not reach significance in young mouse lemurs (r_s_=−0.36, p=0.25, Table 1). The number of errors was not correlated with fasting insulin (r_s_=−0.11, p=0.61, Table 1), HOMA-IR index (r_s_=−0.09, p=0.66, Table 1) or the response to an oral glucose challenge (r_s_=−0.13, p=0.5, Table 1).

The correlation between the number of errors and fasting glucose levels also revealed two subpopulations in middle-aged animals: 44% with normal fasting glycaemia and memory performance similar to young mouse lemur and 56% with impaired fasting glycaemia and memory impairment. These two groups were further identified in a principal component analysis (PCA). The variables discriminating the two subgroups of middle-aged animals were fasting blood glucose (r=0.82, p=1.24 e-7) and number of errors (r=0.76, p=3 e-6) (Supplementary Figure 1).

### Relationships between impaired fasting blood glucose, hippocampus/septum atrophy, and memory performance in middle-aged mouse lemurs

**Figure 4:**
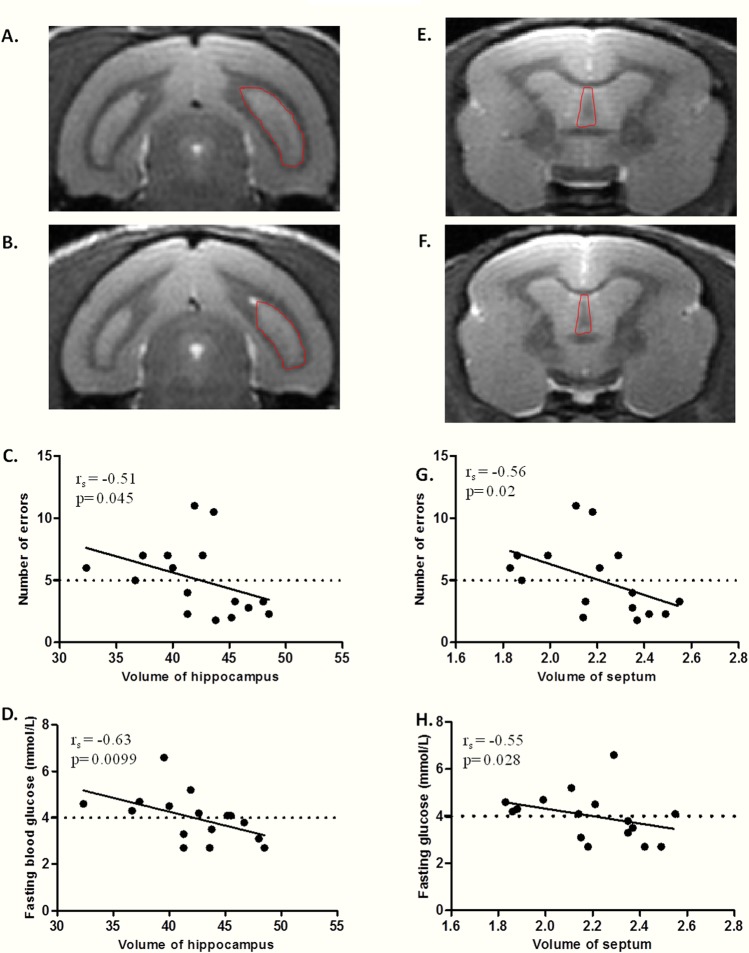
Relationship between fasting blood glucose, hippocampus/septum volume, and spatial memory performance in middle-aged lemurs (**A**-**B**) MRI coronal sections of hippocampus (right hippocampus outlined by dotted line) in a non atrophied middle-aged (**A**) and an atrophied middle-aged (**B**) mouse lemurs. (**C**) Spearman correlation between number of errors and hippocampus volume. (**D**) Spearman correlation between fasting blood glucose and hippocampus volume. (**E**-**F**) MRI coronal sections of septum (outlined by dotted line) in a non atrophied middle-aged (**E**) and an atrophied middle-aged (**F**) animals. (**G**) Spearman correlation between number of errors and septum volume. (**H**) Spearman correlation between fasting blood glucose and septum volume.

Volumes of hippocampus, septum and caudate nucleus were determined in middle-aged mouse lemurs (Figure 4). No significant correlation occurred between regional brain volumes and age or body mass (Table 3).

**Table 3:** Spearman correlation rank coefficients between regional brain volume and physical, memory and glucose homeostasis parameters in middle-aged animals

Region		Hippocampus	Septum	Caudate
Physical conditions	Age	−0.17	−0.40	0.12
	Body mass	−0.08	0.19	0.01
Memory performance	Number of errors.	−0.51*	−0.56*	0.08
Glucose homeostasis	Fasting blood glucose	−0.63**	−0.55*	0.16
	Fasting Insulin	0.14	−0.31	−0.02
	HOMA-IR	−0.15	−0.29	0.1
	Glucose tolerance index	0.27	0.46	0.14

* p<0.05

** p<0.001

HOMA-IR: Homeostasis model assessment of insulin resistance.

A significant negative correlation was observed between the number of errors and the volume of hippocampus (r_s_=−0.51, p=0.045 Figure 4C) or septum (r_s_=−0.56, p=0.02 Figure 4G, Table 3), but not with the volume of the caudate nucleus (r_s_=0.08, p=0.71, Table 3).

Multiple regression tests also highlighted that fasting blood glucose negatively correlated respectively with hippocampus (r_s_=−0.63, p=0.0099; Figure 4D) and septum (r_s_=−0.55, p=0.027; Figure 4H) volumes but not with the volume of the caudate nucleus (r_s_=0.16, p=0.52, Table 3). Hippocampus, septum or caudate nucleus volume did not correlate with fasting insulinemia, HOMA-IR index or glucose tolerance index (Table 3).

**Figure 5:**
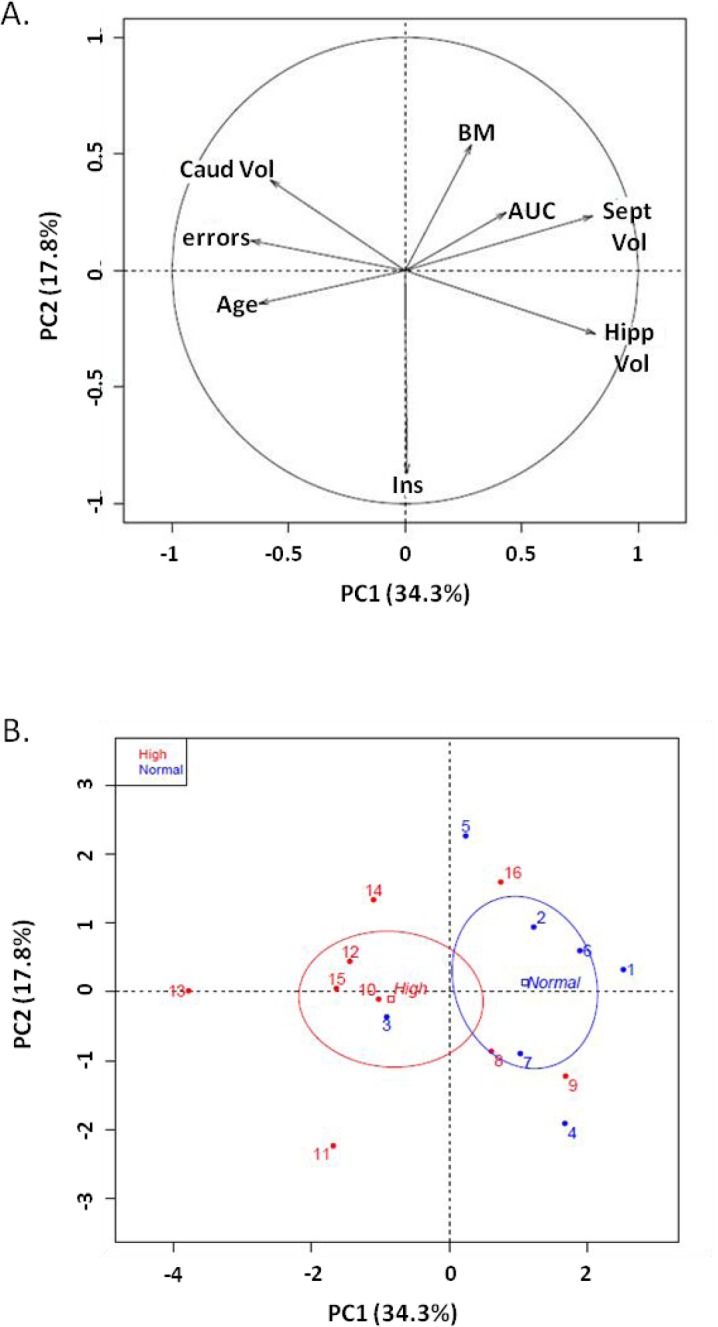
Principal Component Analysis on middle-aged animals (**A**) Variable factor map. Fasting blood glucose was considered as the qualitative variable on age, body mass (BM), number of errors (errors), volumes of hippocampus (Hipp Vol), septum (Sept Vol) and caudate nucleus (Caud Vol), insulin (Ins) and glucose tolerance index (OGTT-AUC) (**B**) Individual dispersion of PCA: x-axis: principal component 1 (PC1: 34.3%), y axis: principal component 2 (PC2: 17.8%). A threshold of 4 mmol/L defined normal blood glucose (fasting glucose less than 4 mmol/L, blue) and high blood glucose (fasting glucose more than 4 mmol/L, red).

Principal component analysis with fasting blood glucose as qualitative variable was performed in the middle-aged group, on age, body mass, insulin, glucose tolerance test, memory performance (number of errors) and regional brain volumes. Individual dispersion represented two groups with principal component 1 (PC1: 34.3%) and principal component 2 (PC2: 17.8%) (Figure 5). The threshold of 4 mmol/L discriminated normal and high glucose levels (Figure 5). Variables discriminating the two groups were regional brain volume: hippocampal volume (r=0.81, p=0.0001), septum volume (r=0.80, p=0.0001) and number of errors (r=−0.66, p=0.005). Similarly, principal component analysis with memory performance as qualitative variable was performed, in the middle-aged group, on age, body mass, glucose parameters and brain region volume. Individual dispersion represented two groups with principal component 1 (PC1: 32.7%) and principal component 2 (PC2: 18.5%) (Supplementary Figure 2). A threshold of 5 errors was used to qualify good and poor performers. Variables discriminating the two groups were brain region volume: hippocampal volume (r=0.86, p=02e-5), septum volume (r=0.83, p=7.03e-5) and fasting glucose levels (r=−0.51, p=0.0045).

## DISCUSSION

Impairment of fasting blood glucose (IFG) is a prediabetic stage that reflects early alteration of glucose metabolism and that may have consequences on health and mortality. Here, we described a clear relationship between fasting blood glucose levels and age in mouse lemur primates but highlighted an heterogeneity in middle-aged animals. Forty four percent of middle-aged animals displayed the same fasting blood glucose levels as young mouse lemurs whereas a majority (56%) presented high normal fasting blood glucose levels, *i.e*. an IFG condition. No significant difference in body weight, insulin resistance (HOMA-IR index) or glucose tolerance was observed between young and middle-aged animals, confirming the prediabetic stage.

Fasting blood glucose was positively correlated to spatial memory impairment in middle-aged animals and with an atrophy of the hippocampus and septum, regions that are well correlated with memory performance. As humans, mouse lemurs display age-related cerebral atrophy and evaluation of regional brain volume was previously compared in young (1.9 to 2.8 years old) and in aged (6.2 to 11.8 years old) mouse lemurs [29–30]. Caudate nucleus atrophy was clearly observed in the oldest animals whereas shrinkage of hippocampus and septum regions was only present in a subgroup of older animals. Herein, we performed MRI analysis in middle-aged animals. The range of caudate nucleus volumes in middle-aged animals was similar to those measured in the young ones [29] suggesting that caudate atrophy is only occurring at very old ages. Heterogeneity of hippocampus and septum volumes was already observed in middle-aged animals. Some animals had a volume similar to young animals whereas others had a volume comparable to old animals [29]. Thus, hippocampus/septum atrophy begins in middle-age while caudate nucleus volume is later affected. Hippocampus and septum are two regions involved in spatial memory abilities [31–32] and a negative correlation was evidenced between hippocampus/septum volume and memory performance in old animals [29]. Here we confirmed this relationship in middle-aged animals.

The origin of cerebral atrophy in lemurs has been explored in previous studies and a relationship between atrophy and intracellular amyloid deposition has been suggested [33], but this study was based on few animals. In humans, IFG is related to poor cognitive performance and to atrophy of hippocampus [16–17, 19, 34]. We thus assessed whether these parameters could be linked to atrophy in lemurs and found that it is probably the main factor leading to pathologic hippocampal/ septal atrophy and the associated cognitive alterations in lemurs. Yet, reverse cause-effect relations are also possible [35].

IFG is a largely developed condition in middle-age healthy human [16–17, 19, 34]. It is related to poor cognitive performance and to atrophy of hippocampus [16–17, 19, 34], and concomitant to neuroinflammatory changes dependent on brain stress and NF-Kb [36]. It is thus critical to further evaluate this condition. Here, we showed that mouse lemurs are critical models to further evaluate this condition, to decipher mechanisms that may link fasting blood glucose levels, cerebral atrophy and cognition and to test therapies.

## METHODS

### Animals

Twenty eight female grey mouse lemurs (*Microcebus murinus*), used in this study were born and reared in the Brunoy colony (MNHN, France, license approval N° E91-114-1). The age of mouse lemurs used in the experiments ranged between 2.4 and 6.1 years old (Table 1). During this study, mouse lemurs were housed in cages in pairs. The cages (50 × 49 × 50 cm) were equipped with wooden branches and wooden nests (nestbox) and kept at standard temperature (24–26°C) and relative humidity (55 %). In the colony, animals were kept in alternating 6-month period of long days (14:10 light/dark, summer-like photoperiod) and short days (10:14 light/dark, winter-like photoperiod). Light was provided by cool fluorescent lamps (250-350 lux) and a dim red light (about 0.002 lux) was provided during the dark phase. All experiments were performed during the long day period. The animals were provided daily (just before the nocturnal phase) with fresh fruits (6 g of banana) and 15 g of a mixture composed of cereal, milk and egg. Water was provided *ad libitum*. Before entering the study, all animals were checked for health. All experiments were ethically reviewed and carried out in accordance with the European Communities Council Directive 86/609/EEC and the GSK Policy on the Care, Welfare and Treatment of Animals under authorization n°91–582 delivered by the “Direction Départementale de la Protection des Populations de l'Essonne”.

### Evaluation of glucose metabolism

***Evaluation of fasting blood glucose and glycemic response to Oral Glucose Tolerance Test (OGTT)*** Glucose metabolism was assessed three months after the onset of the long day season. Fasting glycaemia was measured using a non-invasive method with a hand-held blood glucose meter (Accu-Chek Active®, Hoffmann-La Roche, Switzerland) at the end of the animal resting phase but before food became available. Oral glucose tolerance was tested using a 1.75 g glucose.kg^−1^ body mass glucose challenge, consistent with previous research in rodents [37]. Animals received the oral glucose load at the end of the resting phase of the animal in the form of anhydrous glucose (Glucose Rectapur®, BDH Prolabo, UK) diluted in 0.6 ml of water, administered per os over 1 min. The hand-held glucometer required only 5 μl of total blood for each test stick, which were collected in duplicates. The first measurement corresponded to fasting glycemia. Blood was then sampled at 30, 60 and 120 minutes after the glucose challenge. Glucose tolerance index was defined by Area Under Curve (AUC). The AUC was calculated from the results of OGTT using the trapezoidal method (graph pad prism software). A graph was plotted with time (min) as the horizontal axis and glucose level (mmol/l) as the vertical axis, and the area under the line connecting the four measured values was calculated by multiplying time by glucose level. AUC was expressed in mmol/L.min. We used the fasting glucose of each animal as baseline.

#### Evaluation of fasting blood insulin

Fasting blood sampling for insulin assays was performed at the same time as the sampling for baseline glycaemia. Blood samples were taken via the saphenous vein, without anesthesia, at the end of the resting phase but before food became available. Blood samples were collected in tubes containing EDTA and represented less than 1% of the blood volume of each animal. Blood samples were centrifuged at 2000 g at 4°C for 30 min. Fasting insulin was measured in the plasma using the Human Insulin assay method (Elisa technology, Cat. # EZHI-14K).

#### Insulin resistance index: HOMA-IR

Basal insulin resistance was estimated by the HOMA-IR index, which was calculated using fasting glucose and fasting insulin as an assessment of basal insulin resistance [38]. Fasting insulin and glucose collected at the end of the resting phase were used in the HOMA-IR formula as follows: HOMA-IR = Fasting insulin (μIU/mL) * Fasting glucose (mmol/L) / 22.5 [39].

### Evaluation of spatial reference memory performance by Barnes maze test

The spatial memory performances of female mouse lemurs were evaluated with the Barnes circular maze test, a hippocampus-dependent cognitive task that requires spatial reference memory [27, 40]. The apparatus consisted of a circular platform separated into twelve compartments located at the periphery [27]. Each compartment contained one circular hole giving access to a black Plexiglas nestbox which served as refuge. Fourteen objects (with different forms, sizes and colors) were attached around the periphery of the platform to serve as extra-maze visual cues. Before each trial, the platform and the target box were cleaned, and the platform randomly rotated on its central axis to avoid the use of intra-maze cues.

During the testing procedure, animals experienced one day of habituation and training (day 1) and one day of testing (day 2). Each day comprised four trials, each of which began with placement of the animal inside the starting box at the center of the maze. After one minute, the box was lifted to release the animal. The aim was to reach the target box positioned beneath one of the twelve holes, kept constant in the room for all trials. When the animal entered the target box, the trial was stopped, and the animal remained in the target box for 2 minutes.

On day 1, trials 1 and 2 consisted of placing the animal in a four-walled chamber containing only the open target compartment (one-choice test), which provided access to the target box. For trials 3 and 4, the platform comprised six evenly spaced open compartments (six choices test). These two trials permitted the animal to explore the maze, observe the visual cues and further learn the position of the target box.

On day 2 (testing day), twelve compartments were opened during the four trials. Performances were assessed by the number of errors (*e.g*. the number of entries into compartments containing the non-escape holes) prior to reaching the target box.

The eyes of the lemurs were examined by a veterinary ophthalmologist and no anomalies (e.g. cataract, sclerosis of the lens) were detected that would affect visual acuity.

### Magnetic resonance image acquisition and processing

Brain images were recorded on a 7.0 Tesla spectrometer (Varian) using a four channel phase surface coil (RapidBiomed) actively decoupled from the transmitting birdcage probe (RapidBiomed). Briefly, animals were pre-anesthetized with atropine (0.025 mg/kg subcutaneously) and anaesthetized with isoflurane (4% for induction and 1-1.5% for mainte-nance). Respiratory rate was monitored to insure animal stability until the end of the experiment. Body temperature was maintained by an air-heating system. Two-dimensional fast spin-echo images were recorded with an isotropic nominal resolution of 230μm (128 slices, TR/TE = 10000/17.4 ms; rare factor = 4; acquisition time = 32 min). MR images were zerofilled to reach an apparent isotropic resolution of 115μm.

Morphometric analyses of the brains were performed using Anatomist® software (http://brainvisa.info/index_f.html). Before morphometric analysis, the brain images were rotated so that those for all mice were in a similar orientation. Morphometric analyses were then performed by measuring the volumes of three brain regions (hippocampus, septum and caudate nucleus) in accordance with the stereotaxic brain atlas of the grey mouse lemur by Bons et al. [41], which was used as a reference for all anatomical landmarks. Each structure was manually outlined on coronal sections using a digitizing tablet (Anatomist drawing tools) as described previously [29]. The quality of the delineation of each structure was checked by examination of axial sections. For each animal, the intracranial volume was also measured. The reported volume for each brain structure was normalized by the intracranial volume.

### Data analysis

Data were presented as mean ± SEM. Statistical analysis was performed by Mann Whitney test and Spearman's correlation using graph pad prism software. We used Two way Anova with repeated-measures and Bonferroni tests for post hoc analyses to compare glucose data after oral glucose loading. Principal component analysis and presentation of PCA map were performed using R version 3.2.0. P values less than 0.05 were considered statistically significant.

## SUPPLEMENTARY MATERIAL TABLES


