# Supplementary material for: Impaired fasting blood glucose is associated to cognitive impairment and cerebral atrophy in middle-aged non-human primates
Source: Aging (Albany NY). 2016 Dec 28;9(1):173–84. doi: 10.18632/aging.101148 (PMC5310663; doi:10.18632/aging.101148)
Supplement: Supplementary file 1 [file aging-09-0173-s001.pdf]

SUPPLEMENTARY MATERIAL

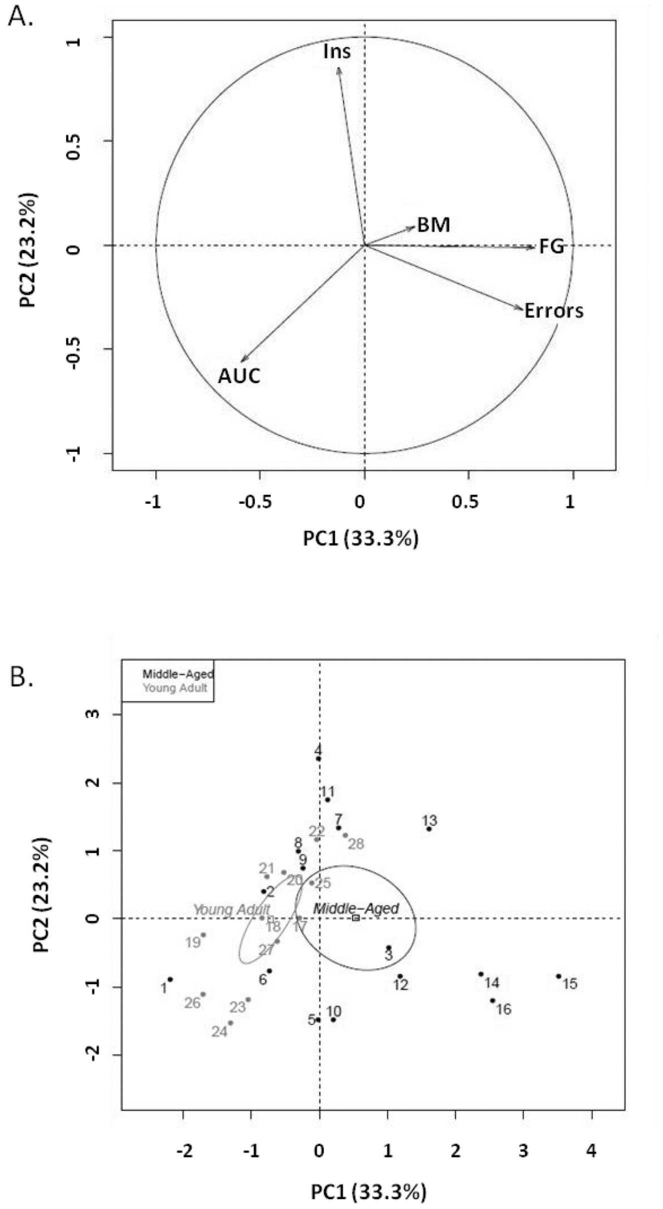

Supplementary Figure 1. PCA on young and middle-aged animals. (**A**) Variable factor map. Age was considered as qualitative variable. Body mass (BM), number of errors (errors), fasting blood glucose (FG), fasting insulinemia (Ins) and glucose tolerance index (AUC). (**B**) Individual dispersion of PCA: x-axis: principal component 1 (PC1: 32.3%), y axis: principal component 2 (PC2: 23.2%). Young animals (grey symbols: range: 2.4 to 3.5 years old) were labeled 17 to 28 and middle-aged animals (black symbols: range: 4.1 to 6.1 years old) were labeled 1 to 16.

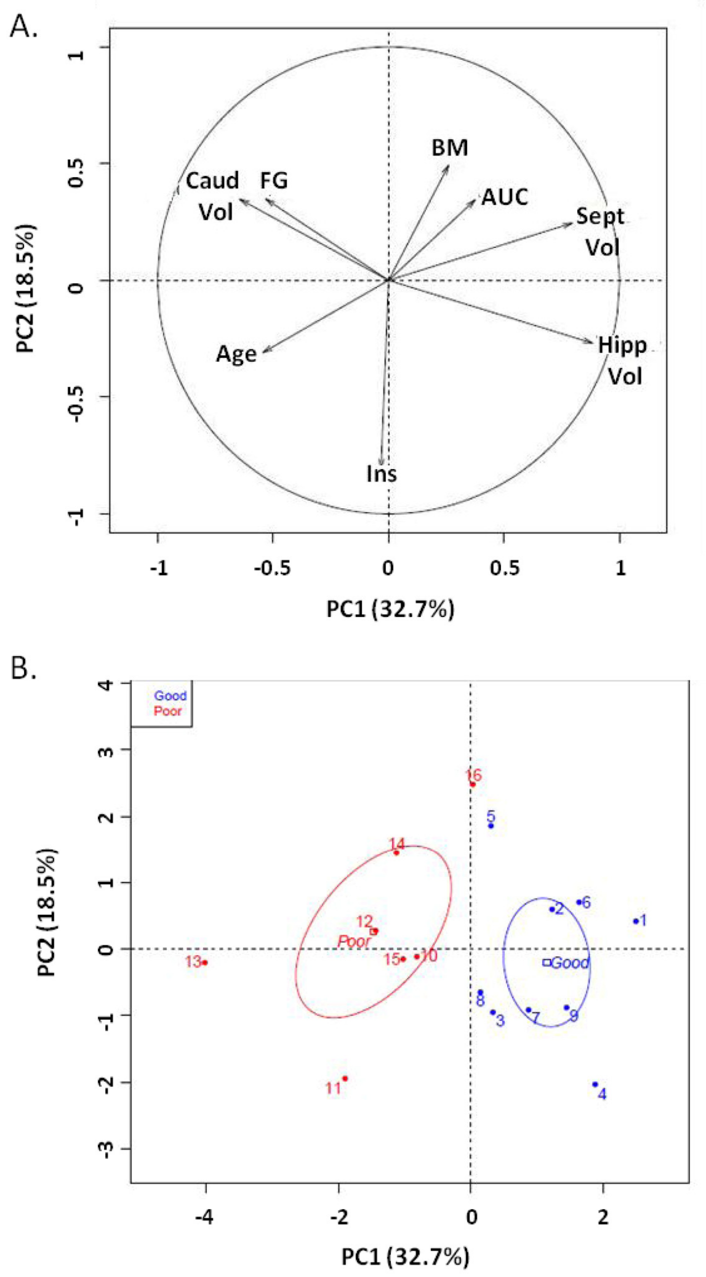

Supplementary Figure 2. PCA on middle-aged animals with number of errors as qualitative variable. (**A**) Variable factor map. Number of errors was considered as qualitative variable. Age, body mass (BM), volume of hippocampus (Hipp Vol), septum (Sept Vol) and caudate nucleus (Caud Vol), fasting blood glucose (FG), insulinemia (Ins) and glucose tolerance index (AUC). (**B**) Individual dispersion of PCA: x-axis: principal component 1 (PC1: 32.7%), y axis: principal component 2 (PC2: 18.5%). A threshold of 5 errors defined two groups: good performers (number of errors inferior to 5, blue) and poor performers (number of errors superior to 5, red).
